# Obesity and sickness absenteeism among health workers in a private hospital in South Africa

**DOI:** 10.4102/safp.v64i1.5418

**Published:** 2022-02-03

**Authors:** Therese de Wet, Willem H. Kruger, Gina Joubert

**Affiliations:** 1 Department of Community Health, Faculty of Health Sciences, University of the Free State, Bloemfontein, South Africa; 2 Kimberley Gariep Mediclinic Hospital, Kimberley, South Africa; 3 Department of Biostatistics, Faculty of Health Sciences, University of the Free State, Bloemfontein, South Africa

**Keywords:** obesity, overweight, BMI, sickness absenteeism, health workers

## Abstract

**Background:**

There is a worldwide trend among the general population including health workers to become more overweight and obese. Such obesity can reduce work ability as manifested through sickness absenteeism. The aim of this study was to describe the obesity among health workers in a private hospital in central South Africa, as measured by the body mass index (BMI) as well as the association of obesity and sickness absenteeism.

**Methods:**

A cohort analytical study was conducted to describe changes in the BMI of employed health workers as well as the association of obesity and absenteeism in a private hospital in South Africa. The BMI measurement on employment, a repeat BMI at the time of the study as well as the sick leave days taken since employment of all health workers who had been employed for more than one year were analysed.

**Results:**

Full time employees (*n* = 344) participated in the study of whom 33.7% were obese; 26.2% were overweight; 36.3% had normal weight and 3.7% were underweight at employment. On repeat BMI done in February 2016, 43.0% were obese; 27.6% were overweight; 28.2% had normal weight and 1.2% were underweight. There was no difference in the amount of sick leaves taken between the normal weight, overweight and obese groups.

**Conclusion:**

A trend among health workers to change to a higher BMI category during employment is concerning, but there was no statistically significant association between the different weight groups and sickness absenteeism. The negative impact of obesity on the productivity of workers cannot be ignored.

## Introduction

Obesity can be considered a disease caused by an unhealthy lifestyle that has reached epidemic proportions.^1^ The statement was confirmed by the World Health Organization (WHO) when it declared obesity as the largest non-communicable disease in adults, which is increasingly turning into a more serious problem than malnutrition.^2^ An et al.^3^ conducted a study across 175 countries and found that the global average adult obesity prevalence steadily increased from 5% in 1975 to 19% in 2016. They also found that a 10% increase in the openness index was associated with an increase in adult obesity prevalence of 1.44% in Asia, but a less strong relationship was found in North American and African countries with a 10% increase in the openness index associated with an increase in adult obesity prevalence of 0.41% and 0.21%, respectively. The body mass index (BMI) is a simple screening tool that is commonly used to classify underweight, normal weight, overweight and obesity in adults and is defined as the weight in kilograms divided by the square of the height in metres (kg/m^2^).^4^ The WHO defines obesity as having a BMI of at least 30.0 kg/m^2^ and can be further subdivided into class I (BMI 30.0 kg/m^2^ – 34.9 kg/m^2^), class II (BMI 35.0 kg/m^2^ – 39.9 kg/m^2^) and class III (BMI ≥ 40.0 kg/m^2^).^5^ However, the limitations of using the BMI to measure obesity should be noted because some factors and circumstances could cause a mismatch between the BMI measurements and true body fatness resulting in misleading information.^6^

The global trend of obesity is also evident in South Africa. Cois and Day^7^ found a statistically significant increase in the average rate of change in BMI between 2008 and 2012. Wagner et al.^8^ conducted a study on rural South African adults and found that the BMI of women rose to 28.7 kg/m^2^ in 2016. In addition, a study conducted in the Southern Africa development community countries found that overweight in women increased by 8.3% from 1990 to 2019 and by 8.5% in men, while in 2019, South Africa (44.7%) had the highest prevalence of obesity in females.^9^ A complicating factor is that the majority of overweight South Africans are unable to correctly identify their own body size as measured by the BMI.^10^ In addition, patients should understand that, because obesity is a chronic disease, weight management will need to be lifelong. Furthermore, one of the most challenging aspects of obesity management relates to the sociocultural environment that could have important implications for the prevalence of overweight and obesity as well as the intervention strategies among African populations. This challenge is evident in studies among African populations that showed high social and cultural valuation of large body size, which could be significant when obesity is viewed as an acceptable norm within a community.^11,12^ Such a challenge faced by obesity prevention programmes is not unique to the African population, but it is less of a problem in other countries.^11^

Overweight and obese individuals experience increased morbidity associated with hypertension, type 2 diabetes, coronary heart problems, respiratory problems and osteoarthritis.^1,13^ The increasing prevalence of obesity has serious health implications, not only for the onset of chronic diseases but also for a higher risk of occupational injury, which is endangering the sustainability of healthcare systems worldwide.^14^ Overweight and obesity can significantly reduce work ability while work ability reflects the balance between work demands and worker capacity.^15^ A study conducted among the general working population indicated that BMIs above the normal range were progressively associated with lower work ability in relation to the physical demands of the job.^16^ Furthermore, it has also been found that work ability is associated with mental health and is therefore compromised among the obese population.^17^ Such obesity-related reduction of work ability can manifest as decreased productivity or as increased frequency of sickness absence spells.^18^

Sickness absenteeism is defined as the time absent from work because of illness.^19^ Fitzgerald et al.^14^ confirmed that obesity is recognised as a significant predictor of sick leave and it has been shown that obese workers experienced higher rates of sickness absenteeism,^20,21^ and presenteeism.^5^ Even though obesity has been associated with significantly greater absenteeism among United States (US) workers, after controlling for demographic characteristics,^5^ the causes of workplace absenteeism are multifaceted and not limited to obesity and health status outcomes.^14^ However, it is stated that living with obesity impairs the quality of life and the ability to function in daily life while it can even increase the risk of psychiatric and affective disorders.^22^ Furthermore, obesity is associated with both single and multimorbidity^23^ as well as permanent work loss, which includes disability pension and premature death.^24^ Therefore, monitoring sickness absenteeism that is linked to excess weight-related conditions provides indirect information about both physical and mental health and is therefore a useful way of evaluating the functional health, including those of health workers.

Health workers are essential professionals whose work is critical to the maintenance of healthy lifestyles and a healthy society while acting as health mentors to the general population.^25^ They have the principal responsibility of encouraging appropriate lifestyle changes that helps to prevent non-communicable diseases, including those associated with obesity.^26^ Obesity among employees in the workplace has shown an alarming rise in prevalence across industries and occupational groups. Some of the main reasons stated are family lifestyle, unhealthy diet and eating habits, and social and economic issues.^5^ Health workers are not excluded from these occupational groups and many of these categories are at risk of developing obesity as a result of their unique work environment and job requirements.^27^ Healthcare systems cannot afford to ignore the impact of obesity on the existing global shortage of health workers. A study conducted in two districts of the Limpopo province of South Africa found that 78% of nurses are overweight or obese.^28^ Another study in the Eastern Cape province reported an alarming rise in the prevalence of abdominal obesity among these professional nurses as high as 90%.^29^ Therefore, the researchers decided to conduct an obesity-related study among the health workers in a hospital environment in central South Africa.

### Aim

The researchers aimed to describe abnormal body mass with a specific focus on obesity among the health workers in a private hospital in the Northern Cape province in South Africa as measured by the BMI as well as the association of obesity and sickness absenteeism.

## Materials and methods

### Study design and setting

A cohort analytical study was conducted among the health workers at a private hospital in Kimberley, Northern Cape, South Africa as the primary researcher is working as the occupational medicine practitioner in the hospital.

### Study population and sampling strategy

The study population included all health workers who worked for at least a period of one year from the date of employment until 28 February 2016 at the mentioned hospital. Kitchen staff and cleaners were excluded because they were contracted from a private company whose health and sickness absenteeism records were not available to the researchers. Based on the exclusion criteria, others who were excluded from the study were the employees who did not have a BMI measured at the start of employment (*n* = 37), pregnant employees (*n* = 5), employees on maternity leave (*n* = 3), employees who refused to participate (*n* = 3), employees on annual leave (*n* = 1), employees on extended sick leave (*n* = 2), employees on training courses (*n* = 1) and employees who had retired after the ethics committee gave the permission to conduct the study (*n* = 4).

### Data collection

The initial BMI measurement by the occupational health nurse when the health worker was employed was obtained from the employee health records at the occupational health clinic of the hospital. After obtaining informed consent, the occupational health nurse and primary researcher measured the repeat BMI of each employee at the follow-up visit who was included in the study during February 2016. The measurement bias was minimised by the fact that the measurements were done by the occupational health nurse and were confirmed by the primary researcher using a calibrated scale. The primary researcher also confirmed the measurements to calculate the BMI of each participant. Furthermore, the standard formula was used to note down the BMI. The occupational health nurse captured the demographic data (age, gender, job title, department, date of employment) of each employee from the health records kept in the occupational health clinic. The data on sickness absenteeism (total sick leave days taken because of injury on duty and sickness since employment, total amount of sick leave incidents) were captured from employees’ leave registers as maintained on the hospital database after permission was obtained from the Human Resource Department of the hospital.

For analysis the BMI was categorised as follows:

- Underweight: BMI < 18.5 kg/m^2^
- Normal range: BMI 18.5 kg/m^2^ – 24.99 kg/m^2^
- Overweight: BMI 25.00 kg/m^2^ – 29.99 kg/m^2^
- Obese: BMI ≥ 30 kg/m^2^ with categories class I (BMI 30.0 kg/m^2^ – 34.9 kg/m^2^), class II (BMI 35.0 kg/m^2^ – 39.9 kg/m^2^) and class III (BMI ≥ 40.0 kg/m^2^)^4^

### Data analysis

Sick days and incidents (the number of times a health worker took leave using sickness as a reason) were expressed per year to enable comparison between persons with varying length of employment. Data were analysed by the Department of Biostatistics, Faculty of Health Sciences at the University of the Free State (UFS). The results were summarised by frequencies and percentages (categorical variables) and medians and interquartile ranges (IQR) (numerical variables, because of skew distributions). Subgroups were compared using non-parametric Kruskall-Wallis tests with significance level set at 0.05. All analyses were performed using SAS version 9.4.

### Pilot study

A pilot study was conducted on seven employees after ethics approval was given and no changes were made. During the pilot study, the researchers realised that some employees did not have a BMI done on employment and they were excluded from the main study.

## Results

The hospital had 502 full-time employees who qualified for the study and 344 (68.5%) agreed to participate. Socio-demographic details are presented in Table 1. The median age of the participants was 37.8 years (IQR: 31.3–46.8 years). The median age of participating administrative staff was 38.5 years (IQR: 31.8–47.0 years) and of participating nursing staff 37.3 years (IQR: 31.2–46.6 years). The participants were administrative (42.2%) and nursing personnel (57.8%), and predominantly women (76.5%).

**TABLE 1:** Socio-demographic information (*n* = 344).

Variable	All participants (*n* = 344)		Nursing staff (*n* = 199)		Administrative staff (*n* = 145)	
	*n*	%	*n*	%	*n*	%
**Gender**						
Female	263	76.5	162	81.4	101	69.7
Male	81	23.5	37	18.6	44	30.3
**Department**						
Wards	109	31.7	-	-	-	-
Administrative offices	76	22.1	-	-	-	-
Theatres	42	12.2	-	-	-	-
Critical care	32	9.3	-	-	-	-
Sterilisation and housekeeping	21	6.1	-	-	-	-
Pharmacy	19	5.5	-	-	-	-
Training	19	5.5	-	-	-	-
Emergency and clinic	14	4.1	-	-	-	-
Technical services	12	3.5	-	-	-	-

Table 2 categorises participants according to their BMI status at the start of employment and at the follow-up measurement visit. At the start of their employment, 33.7% of the participants were obese, 26.2% were overweight, 36.3% had a normal weight and 3.7% were underweight. During the follow-up BMI measurement visit in February 2016, it was found that 43.0% of the participants were obese, 27.6% were overweight, 28.2% had a normal weight and 1.2% were underweight.

**TABLE 2:** Body mass index at the start of employment and body mass index at the time of the study.

BMI classification	BMI at the start of employment		BMI at follow-up measurement visit	
	*n*	%	*n*	%
**All participants (*n* = 344)**				
Underweight	13	3.8	4	1.2
Normal	125	36.3	97	28.2
Overweight	90	26.2	95	27.6
Class I obesity	58	16.9	69	20.1
Class II obesity	34	10.0	30	8.7
Class III obesity	24	7.0	49	14.2
**Nursing staff (*n* = 199)**				
Underweight	8	4.0	1	0.5
Normal	68	34.2	54	27.1
Overweight	52	26.1	54	27.1
Class I obesity	33	16.6	39	19.6
Class II obesity	23	11.6	33	16.6
Class III obesity	15	7.5	49	14.2
**Administrative staff (*n* = 145)**				
Underweight	5	3.5	3	2.1
Normal	57	39.3	43	29.7
Overweight	38	26.2	41	28.3
Class I obesity	25	17.2	30	20.7
Class II obesity	11	7.6	12	8.3
Class III obesity	9	6.2	16	11.0

BMI, body mass index.

Table 3 indicates the changes of the BMI status of participants from the start of employment to the follow-up measurement visit. The majority (70.7%) of all participants (68.3% of nursing staff and 73.8% of administrative staff) were in the same BMI category at both time points whereas 25.4% of all participants (26.6% of nursing staff and 23.4% of administrative staff) were in a higher BMI status category at follow-up.

**TABLE 3:** Comparison of body mass index at the start of employment and body mass index at the time of the study.

BMI at start of employment	BMI at follow-up measurement visit			
	Underweight (%)	Normal weight (%)	Overweight (%)	Obese (%)
**All participants (*n* = 344)**				
Underweight	0.9	2.6	0.3	0.0
Normal weight	0.3	23.6	11.1	1.5
Overweight	0.0	1.7	14.5	9.9
Obese	0.0	0.3	1.7	31.7
**Nursing staff (*n* = 199)**				
Underweight	0.5	3.5	0.0	0.0
Normal weight	0.0	20.6	11.1	2.5
Overweight	0.0	2.5	14.1	9.6
Obese	0.0	0.5	2.0	33.2
**Administrative staff (*n* = 145)**				
Underweight	1.4	1.4	0.7	0.0
Normal weight	0.7	27.6	11.0	0.0
Overweight	0.0	0.7	15.2	10.3
Obese	0.0	0.0	1.4	29.7

BMI, body mass index.

Table 4 shows that the median employment years for the group was 6.8 years and the median sick days taken per year was 4.5 days.

**TABLE 4:** Employment duration and sickness characteristics.

Variable	Median	Interquartile range
**All participants (*n* = 344)**		
Employment duration (years)	6.8	3.1–11.6
Number of sick days taken per year	4.5	2.2–8.1
Number of sick leave incidents per year	1.5	0.8–2.7
Number of injury on duty cases per year	0.0	0.0
**Nursing staff (*n* = 199)**		
Employment duration (years)	6.0	2.8–11.3
Number of sick days taken per year	4.3	2.3–8.2
Number of sick leave incidents per year	1.7	0.9–2.7
Number of injury on duty cases per year	0.0	0.0
**Administrative staff (*n* = 145)**		
Employment duration (years)	7.4	3.6–12.6
Number of sick days taken per year	4.8	2.2–8.1
Number of sick leave incidents per year	1.5	0.8–2.9
Number of injury on duty cases per year	0.0	0.0

Table 5 summarises the demographic and sick leave characteristics of the main BMI categories. The age and total time in employment differed significantly (*p* < 0.01) between these groups, with those who started overweight or obese and were obese at follow-up being significantly older and longer in employment. However, there was no significant difference in sick leave days taken per year (*p* = 0.96) between the main BMI categories. The below analysis was carried out separately for administrative and nursing staff and the same patterns were observed.

**TABLE 5:** The association between body mass index and sickness absenteeism (*n* = 312).

BMI cross-classification category	*n*	Variable	Median	Interquartile range
Normal BMI stayed normal	81	Age (years)	32.1	28.9–40.3
		Sick days per year	4.2	2.2–7.9
		Total time employed in years	4.5	2.7–8.6
Normal BMI became overweight	38	Age (years)	34.3	31.0–40.8
		Sick days per year	4.2	1.8–8.5
		Total time employed in years	5.3	2.8–11.1
Overweight BMI stayed overweight	50	Age (years)	38.1	30.6–46.9
		Sick days per year	4.6	1.5–7.4
		Total time employed in years	4.1	2.2–10.1
Overweight BMI became obese	36	Age (years)	41.1	32.1–49.1
		Sick days per year	5.1	1.6–8.6
		Total time employed in years	9.6	5.2–15.0
Obese BMI stayed obese	109	Age (years)	42.8	35.8–49.8
		Sick days per year	4.1	2.4–9.1
		Total time employed in years	8.0	4.0–12.3

BMI, body mass index.

## Discussion

The goal of this research study was to describe abnormal body mass as measured by the BMI among health workers. The results indicated that only 28.2% of health workers in the private hospital had a normal BMI at the time of the study in 2016. The high prevalence of obesity among health workers found in this study is a cause for concern as research has shown the strong association between obesity and a range of adverse health outcomes and obesity-related comorbidities.^30^ The high prevalence of obesity in the current study is evident in that almost 60% of the participants were overweight (27.6%) or obese (43.0%), which is much higher than the general population in South Africa in 2016 with an overweight prevalence of 23.6% and an obesity prevalence of 26.2%.^31^ The reasons for the high obesity rate in the current study was not investigated, but there are several models for the causality of obesity described in the literature.^32^ However, it should be highlighted that the obesity prevention and treatment strategies have not been successful^33^ as evident in the high rate of obesity. A study conducted in Kenya^34^ had similar results with 58.8% of health workers being overweight or obese. In a tertiary hospital in Nigeria^35^, almost 63.4% of the health workers had abnormal BMIs, but more health workers were overweight (31.4%) than obese (23.2%). The study of Kasu et al.^36^ conducted in Ghana also found that more health workers were overweight (25.3%) than obese (12.7%). In contrast, health workers in a study in Malaysia^37^ had a lower prevalence of abnormal BMIs (49.9%) with 28.4% being overweight and 21.5% obesity. The results of the current study cannot be ignored because of the effect of obesity on the productivity of health workers as well as the critical shortage of health workers.^38^

Considering the negative effect obesity has on the health status of people, it is also important to review the trend of obesity that are being experienced worldwide and especially in Africa. The NCD Risk Factor Collaboration (NCD-RisC) – Africa Working Group^39^ highlighted these trends and showed an increase in the mean BMI since 1980 across all regions in Africa. This study confirmed the trend, even though only health workers were included in this study, with approximately a quarter (25.3%) of the employees with a median employment duration of 6.8 years in a higher BMI category than at the start of their employment. Of particular concern was the increase of employees in the obese category (33.7% at the start of employment to 43.0% at the time of the study). Another alarming result is the decrease found in the number of employees in the normal weight category where the percentage decreased from 36.2% to 28.2%. These trends among the participants of this study were realised over a relatively short period of time. Looking at the long-term historical trends in obesity, Chooi et al.^40^ indicated that in the African region overweight and obesity have approximately doubled from 1980 to 2015, namely from 18.5% to 34.5% and from 6.2% to 12.7%, respectively. The trends further indicated that the prevalence of overweight in South Africa increased from 49.4% in 1980 to 57.8% in 2015. The changes in the BMI among the participants in this study over such a short period along with the trends described in the literature cannot be ignored when the urgency to plan and implement preventative programmes to address obesity in South Africa is considered.

Amiri and Behnezhad^41^ conducted a systematic review indicating that overweight and obesity were both a risk factor for sick leave. Therefore, another goal of this study was to describe the association between obesity and sickness absenteeism among the participating health workers. The results showed that the median sick leave days taken for all the participants were 4.5 days per year, which was almost two days less than what was found in a study among health workers in Nigeria.^42^ Roos et al.^43^ mentioned that in previous research both smoking and obesity had been identified as separate risk factors for long spells of sickness absenteeism. However, the current study did not explore smoking as a possible cause for the sickness absenteeism because the data was not available, but the negative health effects of smoking should not be ignored. Virtanen et al.^44^ confirmed the risk to take sick leave days because of obesity when their analyses examined lifestyle as a predictor of sickness absence among employees on sick leave, and found that obesity, along with low physical activity, were associated with longer or more frequent absences and multiple-cause absences at follow-up. Another study also reported that participants with obesity had a higher risk of sickness absence.^45^

However, the current study found that there was no significant difference in sick leave days taken per year among employees who have changed to a higher BMI category. The same pattern was observed when the participants were put in the various categories of health workers. An explanation for this could be that the study was conducted in a private hospital, and that more stringent measures are in place to manage sick leave. However, absenteeism is a complex human resource matter. It is an inadequate response of individual workers to their work environment, which is a reflection of the employee’s job dissatisfaction.^46^ The above-mentioned non-significant finding is in contradiction to other studies conducted that concluded that sick absence gradually increased with an increase in BMI.^20,47^ Roos et al.^48^ also confirmed that obesity increased the risk of both self and medically certified sickness absence during weight changes. In contrast, a systematic review concluded that there is ‘inconclusive evidence’ for overweight to be related to either long-term or short-term sick leave, but it should be noted that this conclusion is drawn because of the inconsistent results observed regarding the lack of statistically significant differences in sick leave between overweight workers and normal-weight workers.^49^ It was also found that excess short-term sickness absence could not be explained by obesity-related medical problems.^50^ The current study did not differentiate between the various reasons or diagnosis for taking sick leave despite the importance thereof. However, the researchers agree with the recommendation by Svärd et al.^47^ that the diagnosis for sick absence needs to be studied in order to understand the association between BMI, health and work disability better.

## Limitations

In the current study, only the BMI was used as an anthropometric measure, while abdominal obesity is another entity to consider when evaluating health risks related to obesity as the risks may differ as measured by the BMI.^51^ However, it should be noted that there are many indicators that can be used to determine body fat distribution, such as BMI as used in this study. Such other indicators recommended are waist circumference, visceral adipose index, body fat percentage and waist-to-height ratio. Another limitation is that the data only included a baseline BMI done by only the occupational health nurse (no reviewer) and one follow-up BMI with no other BMI measurements over time. It is important to note that the healthy worker effect should be considered as a confounder and selection bias with the interpretation of the results as suggested by Shan,^52^ for example, those who are morbidly obese and unwell may be so sick that they were not at work during the time of the study. This current study also did not explore the health risks associated with obesity and therefore it was not possible to identify any causality between specific obesity-related diseases and the health workers using sick leave days. The current study was conducted among health workers in one private hospital in South Africa with a specific leave policy to manage all types of leave. Other hospitals might have different leave policies, and therefore, the results of the current study do not reflect the obesity among health workers working in other types of healthcare institutions. Furthermore, the actual productivity of health workers was not evaluated in this study.

## Recommendations

The high prevalence of overweight and obesity among health workers as well as the rate of changes to a higher BMI during employment found in this study are a matter of concern. Obesity is a complex health-related condition, and there is no single or simple solution to the obesity epidemic. A multifaceted approach is needed, and the workplace is a good starting point. Occupational health clinics and Human Resource Departments should ensure that practical workplace health promotion programmes are in place to emphasise a lifestyle that includes healthy eating and regular physical activity. A supportive environment should be created to promote healthy living behaviours that prevent obesity. Because of the comorbidities associated with obesity, occupational health clinics should have programmes in place to diagnose and manage those comorbidities effectively. The Human Resource Department should consider offering various options to address obesity in the workplace, such as (1) having a policy that prohibits discrimination based on weight, (2) inclusive wellness programmes that should not be used to shame anyone, (3) discounted gym memberships, (4) establishing support groups as a way to help employees connect with others who are facing similar obesity challenges, and (5) conducting job analyses of employees in an attempt to determine the best way to redesign jobs to help reduce obesity.^53^ As stated earlier, this study was only conducted in a private hospital, and additional research, especially in the public sector, should be conducted. Qualitative research could provide more in-depth knowledge as to the reasons for the outcome of this study. Furthermore, health workers should act as role models for the general population when it comes to health promotion regarding weight-related health issues.

## Conclusion

There was a trend among the health workers who participated in this study to change to a higher BMI category during their employment period with a quarter of the employees changing BMI categories for reasons which are unclear/unknown. This is in line with trends worldwide and it is a major cause for concern from a public health perspective. The results of this study did not confirm the results of other studies because no association was found between obesity and absenteeism among various categories of health workers in a private hospital. However, there is a significant difference between the age in the different weight categories as well as the time in years worked in the hospital.

It was not possible with the current study to explore the health risks associated with obesity. Therefore, any causality between specific obesity-related diseases and the health workers using sick leave days was not identified. Further research is needed to determine such health risk factors and causality in South Africa. Even though this study did not find any significant associations between obesity and absenteeism, there is enough evidence in the literature that have shown such associations. In addition, the literature indicated that obesity and its health-related effects could have a major impact on the productivity of health workers. Therefore, considering the outcome of this study, the impact of obesity among health workers on their work productivity and sickness absenteeism cannot be ignored.

## References

[1] Atawia RT, Bunch KL, Toque HA, Caldwell RB, Caldwell RW. Mechanisms of obesity-induced metabolic and vascular dysfunctions. Front Biosci (Landmark Ed). 2019;24(5):890–934. 10.2741/4758 30844720 PMC6689231

[2] Yumuk V, Tsigos C, Fried M, et al. European guidelines for obesity management in adults [published correction appears in Obes Facts 2016;9:64]. Obes Facts. 2015;8(6):402–424. 10.1159/000442721 26641646 PMC5644856

[3] An R, Guan C, Liu J, Chen N, Clarke C. Trade openness and the obesity epidemic: A cross-national study of 175 countries during 1975–2016. Ann Epidemiol. 2019;37:31–36. 10.1016/j.annepidem.2019.07.002 31399309

[4] Juni MH. Obesity: A public health threats in developing countries [Editorial]. Int J Public Health Clin Sci. 2015;2:iv–vi.

[5] Kudel I, Huang JC, Ganguly R. Impact of obesity on work productivity in different US occupations: Analysis of the National Health and Wellness Survey 2014 to 2015. J Occup Environ Med. 2018;60(1):6–11. 10.1097/JOM.0000000000001144 29065062 PMC5770108

[6] Prentice AM, Jebb SA. Beyond body mass index. Obes Rev. 2001;2(3):141–147. 10.1046/j.1467-789x.2001.00031.x 12120099

[7] Cois A, Day C. Obesity trends and risk factors in the South African adult population. BMC Obes. 2015;2:42. 10.1186/s40608-015-0072-2 26617987 PMC4603579

[8] Wagner RG, Crowther NJ, Gómez-Olivé FX, et al. Sociodemographic, socioeconomic, clinical and behavioural predictors of body mass index vary by sex in rural South African adults-findings from the AWI-Gen study. Glob Health Action. 2018;11(sup2):1549436. 10.1080/16549716.2018.1549436 30499746 PMC6282437

[9] Gona PN, Kimokoti RW, Gona CM, et al. Changes in body mass index, obesity, and overweight in Southern Africa development countries, 1990 to 2019: Findings from the Global Burden of Disease, Injuries, and Risk Factors Study. Obes Sci Pract. 2021;7(5):509–524. 10.1002/osp4.519 34631130 PMC8488455

[10] Mchiza ZJ, Labadarios D, Parker W, Bikitsha N. Body-image perception and dissatisfaction: Acknowledging the sociocultural factors of obesity in South Africa. Pretoria: Human Sciences Research Council; 2016 [cited 2021 Feb 26]. Available from: http://hdl.handle.net/20.500.11910/10159

[11] Holdsworth M, Gartner A, Landais E, Maire B, Delpeuch F. Perceptions of healthy and desirable body size in urban Senegalese women. Int J Obes Relat Metab Disord. 2004;28(12):1561–1568. 10.1038/sj.ijo.0802739 15278107

[12] Appiah CA, Steiner-Asiedu M, Otoo GE. Predictors of overweight/obesity in urban Ghanaian women. Int J Clin Nutr. 2014;2(3):60–68.

[13] Xia JY, Lloyd-Jones DM, Khan SS. Association of body mass index with mortality in cardiovascular disease: New insights into the obesity paradox from multiple perspectives. Trends Cardiovasc Med. 2019;29(4):220–225. 10.1016/j.tcm.2018.08.006 30172579

[14] Fitzgerald S, Kirby A, Murphy A, Geaney F. Obesity, diet quality and absenteeism in a working population. Public Health Nutr. 2016;19(18):3287–3295. 10.1017/S1368980016001269 27230727 PMC5197930

[15] Tonnon SC, Robroek SR, Van der Beek AJ, et al. Physical workload and obesity have a synergistic effect on work ability among construction workers. Int Arch Occup Environ Health. 2019;92(6):855–864. 10.1007/s00420-019-01422-7 30941545

[16] Andersen LL, Izquierdo M, Sundstrup E. Overweight and obesity are progressively associated with lower work ability in the general working population: Cross-sectional study among 10,000 adults. Int Arch Occup Environ Health. 2017;90(8):779–787. 10.1007/s00420-017-1240-0l 28660321

[17] Köhler H, Markov V, Watschke A, et al. Psychosocial predictors of work ability in morbidly obese patients: Results of a cross-sectional study in the context of bariatric surgery. Obes Facts. 2021;14(1):56–63. 10.1159/000511735 33352562 PMC7983534

[18] Van den Berg S, Burdorf A, Robroek SJ. Associations between common diseases and work ability and sick leave among health care workers. Int Arch Occup Environ Health. 2017;90(7):685–693. 10.1007/s00420-017-1231-1 28550420 PMC5583264

[19] Yıldız H, Yıldız B, Zehir C, Aykaç M. The antecedents of presenteeism and sickness absenteeism: A research in Turkish health sector. Procedia Soc Behav Sci. 2015;207:398–403. 10.1016/j.sbspro.2015.10.109

[20] Reber KC, König HH, Hajek A. Obesity and sickness absence: Results from a longitudinal nationally representative sample from Germany. BMJ Open. 2018;8(6):e019839. 10.1136/bmjopen-2017-019839 PMC6009453 29880564

[21] Pihlajamäki M, Uitti J, Arola H, et al. Self-reported health problems and obesity predict sickness absence during a 12-month follow-up: A prospective cohort study in 21 608 employees from different industries. BMJ Open. 2019;9(10):e025967. 10.1136/bmjopen-2018-025967 PMC6830705 31676640

[22] Vallis M. Quality of life and psychological well-being in obesity management: Improving the odds of success by managing distress. Int J Clin Pract. 2016;70(3):196–205. 10.1111/ijcp.12765 26842304 PMC5067635

[23] Alaba O, Chola L. The social determinants of multimorbidity in South Africa. Int J Equity Health. 2013;12(1):1–10. 10.1186/1475-9276-12-63 23962055 PMC3846856

[24] Goettler A, Grosse A, Sonntag D. Productivity loss due to overweight and obesity: A systematic review of indirect costs. BMJ Open. 2017;7(10):e014632. 10.1136/bmjopen-2016-014632 PMC5640019 28982806

[25] Ajeani J, Mangwi Ayiasi R, Tetui M, et al. A cascade model of mentorship for frontline health workers in rural health facilities in Eastern Uganda: Processes, achievements and lessons. Glob Health Action. 2017;10(sup4):1345497. 10.1080/16549716.2017.1345497 28816629 PMC5645691

[26] Osei-Yeboah J, Kye-Amoah KK, Owiredu WK, et al. Cardiometabolic risk factors among healthcare workers: A cross-sectional study at the Sefwi-Wiawso Municipal Hospital, Ghana. Biomed Res Int. 2018;2018:8904548. 10.1155/2018/8904548 29850585 PMC5937588

[27] Upadhyaya M, Sharma S, Pompeii LA, Sianez M, Morgan RO. Obesity prevention worksite wellness interventions for health care workers: A narrative review. Workplace Health Saf. 2020;68(1):32–49. 10.1177/2165079919863082 31451058

[28] Goon DT, Maputle MS, Olukoga A, Lebese R, Khoza LB, Ayanwu FC. Overweight, obesity and underweight in nurses in Vhembe and Capricorn districts, Limpopo. S Afr J Clin Nutr. 2013;26(3):147–149. 10.1080/16070658.2013.11734459

[29] Monakali S, Ter Goon D, Seekoe E, Owolabi EO. Prevalence and factors associated with abdominal obesity among primary health care professional nurses in Eastern Cape, South Africa. S Afr Fam Pract. 2018;60(5):146–150. 10.1080/20786190.2018.1467181 PMC6295957 30551716

[30] Shrestha N, Pedisic Z, Neil-Sztramko S, Kukkonen-Harjula KT, Hermans V. The impact of obesity in the workplace: A review of contributing factors, consequences and potential solutions. Curr Obes Rep. 2016;5(3):344–360. 10.1007/s13679-016-0227-6 27447869

[31] World Obesity. Global Obesity Observatory. South Africa. Obesity prevalence. Adults, 2016. London: World Obesity; 2016 [cited 2021 Feb 26]. Available from: https://data.worldobesity.org/country/south-africa-197/#data_prevalence

[32] Frood S, Johnston LM, Matteson CL, Finegood DT. Obesity, complexity, and the role of the health system. Curr Obes Rep. 2013;2(4):320–326. 10.1007/s13679-013-0072-9 24273701 PMC3824226

[33] Blüher M. Obesity: Global epidemiology and pathogenesis. Nat Rev Endocrinol. 2019;15(5):288–298. 10.1038/s41574-019-0176-8 30814686

[34] Ondicho ZM, Omondi DO, Onyango AC. Prevalence and socio-demographic factors associated with overweight and obesity among healthcare workers in Kisumu East Sub-County, Kenya. Am J Med Med Sci. 2016;6(3):66–72.

[35] Dankyau M, Shu’aibu JA, Oyebanji AE, Mamven OV. Prevalence and correlates of obesity and overweight in healthcare workers at a tertiary hospital. J Med Trop. 2016;18(2):55–59. 10.4103/2276-7096.188533

[36] Kasu ES, Ayim A, Tampouri J. Prevalence of obesity among health workers in Kadjebi District of Ghana. J Biol Agr Healthc. 2015;5:155–166.

[37] Kit LP, Saad HA, Jamaluddin R, Phing CH. Prevalence of overweight and obesity among primary healthcare workers in Perak, Malaysia. Int Med J Malays. 2020;19(1):23–30. 10.31436/imjm.v19i1.1327

[38] Maphumulo WT, Bhengu BR. Challenges of quality improvement in the healthcare of South Africa post-apartheid: A critical review. Curationis. 2019;42(1):e1–e9. 10.4102/curationis.v42i1.1901 PMC6556866 31170800

[39] NCD Risk Factor Collaboration (NCD-RisC) – Africa Working Group. Trends in obesity and diabetes across Africa from 1980 to 2014: An analysis of pooled population-based studies. Int J Epidemiol. 2017;46(5):1421–1432. 10.1093/ije/dyx078 28582528 PMC5837192

[40] Chooi YC, Ding C, Magkos F. The epidemiology of obesity. Metabolism. 2019;92:6–10. 10.1016/j.metabol.2018.09.005 30253139

[41] Amiri S, Behnezhad S. Body mass index and risk of sick leave: A systematic review and meta-analysis. Clin Obes. 2019;9(6):e12334. 10.1111/cob.12334 31368657

[42] Oche MO, Oladigbolu RA, Ango JT, Okafoagu NC, Ango UM. Work absenteeism amongst health care workers in a tertiary health institution in Sokoto, Nigeria. J Adv Med Med Res. 2018;26(2):1–9. 10.9734/JAMMR/2018/40467

[43] Roos E, Lallukka T, Lahelma E, Rahkonen O. The joint associations of smoking and obesity with subsequent short and long sickness absence: A five year follow-up study with register-linkage. BMC Public Health. 2017;17(1):978. 10.1186/s12889-017-4997-x 29282110 PMC5745910

[44] Virtanen M, Ervasti J, Head J, et al. Lifestyle factors and risk of sickness absence from work: A multicohort study. Lancet Public Health. 2018;3(11):e545–e554. 10.1016/S2468-2667(18)30201-9 30409406 PMC6220357

[45] Bramming M, Jørgensen MB, Christensen AI, Lau CJ, Egan KK, Tolstrup JS. BMI and labor market participation: A cohort study of transitions between work, unemployment, and sickness absence. Obesity (Silver Spring). 2019;27(10):1703–1710. 10.1002/oby.22578 31544342

[46] Taylor P, Cunningham I, Newsome K, Scholarios D. ‘Too scared to go sick’ – Reformulating the research agenda on sickness absence. Ind Relat J. 2010;41(4):270–288. 10.1111/j.1468-2338.2010.00569

[47] Svärd A, Lahti J, Mänty M, et al. Weight change among normal weight, overweight and obese employees and subsequent diagnosis-specific sickness absence: A register-linked follow-up study. Scand J Public Health. 2020;48(2):155–163. 10.1177/1403494818802990 30269682 PMC7042496

[48] Roos E, Laaksonen M, Rahkonen O, Lahelma E, Lallukka T. Weight change and sickness absence – A prospective study among middle-aged employees. Eur J Public Health. 2015;25(2):263–267. 10.1093/eurpub/cku087 24997201

[49] Van Duijvenbode DC, Hoozemans MJ, Van Poppel MN, Proper KI. The relationship between overweight and obesity, and sick leave: A systematic review. Int J Obes (Lond). 2009;33(8):807–816. 10.1038/ijo.2009.121 19528969

[50] Harvey SB, Glozier N, Carlton O, et al. Obesity and sickness absence: Results from the CHAP study. Occup Med (Lond). 2010;60(5):362–368. 10.1093/occmed/kqq031 20308262

[51] Ma S, Xi B, Yang L, Sun J, Zhao M, Bovet P. Trends in the prevalence of overweight, obesity, and abdominal obesity among Chinese adults between 1993 and 2015. Int J Obes (Lond). 2021;45(2):427–437. 10.1038/s41366-020-00698-x 33037330

[52] Shah D. Healthy worker effect phenomenon. Indian J Occup Environ Med. 2009;13(2):77–79. 10.4103/0019-5278.55123 20386623 PMC2847330

[53] Prieto LC, Mathur-Helm B, Dawson KN. The ethic of care: An HR strategy to address obesity in the workplace. Hum Resour Manag Int Dig. 2018;26(2):12–15. 10.1108/HRMID-07-2017-0131

